# Polymersomes Based Versatile Nanoplatforms for Controlled Drug Delivery and Imaging

**DOI:** 10.34172/apb.2023.028

**Published:** 2022-01-08

**Authors:** Rohini Kotha, Divya Dhatri Kara, Rajeshwari Roychowdhury, Katikala Tanvi, Mahalaxmi Rathnanand

**Affiliations:** ^1^Department of Pharmaceutics, Manipal College of Pharmaceutical Sciences, Manipal Academy of Higher Education (MAHE), Manipal-576104, India.; ^2^Manipal College of Pharmaceutical Sciences, Manipal Academy of Higher Education (MAHE), Manipal-576104, India.

**Keywords:** Polymersomes, Nanotechnology, Film hydration, Double emulsion technique, Microfluidic technique, Therapeutic drug delivery

## Abstract

Drug delivery systems made based on nanotechnology represent a novel drug carrier system that can change the face of therapeutics and diagnosis. Among all the available nanoforms polymersomes have wider applications due to their unique characteristic features like drug loading carriers for both hydrophilic and hydrophobic drugs, excellent biocompatibility, biodegradability, longer shelf life in the bloodstream and ease of surface modification by ligands. Polymersomes are defined as the artificial vesicles which are enclosed in a central aqueous cavity which are composed of self-assembly with a block of amphiphilic copolymer. Various techniques like film rehydration, direct hydration, nanoprecipitation, double emulsion technique and microfluidic technique are mostly used in formulating polymersomes employing different polymers like PEO-b-PLA, poly (fumaric/sebacic acid), poly(N-isopropylacrylamide) (PNIPAM), poly (dimethylsiloxane) (PDMS), and poly(butadiene) (PBD), PTMC-b-PGA (poly (dimethyl aminoethyl methacrylate)-b-poly(l-glutamic acid)) etc. Polymersomes have been extensively considered for the conveyance of therapeutic agents for diagnosis, targeting, treatment of cancer, diabetes etc. This review focuses on a comprehensive description of polymersomes with suitable case studies under the following headings: chemical structure, polymers used in the formulation, formulation methods, characterization methods and their application in the therapeutic, and medicinal filed.

## Introduction

 Recent advancements in the field of biomedicine have resulted in the multifold increase in the development of new formulations to efficaciously treat critical health conditions like cancer, diabetes etc. Problems such as enzyme instability, lack of drug targeting and low stability associated with these new formulations minimize their applications in the biomedical field.^1,2^ Nanotechnology is a multidisciplinary scientific domain that applies molecular engineering and manufactures principles in the production of functional systems of distinctive optical, electronic and physical features that have been used to improve the above drawbacks in the field of diagnosis, treatment and prevention of multifarious diseases in the biomedical sector.^3,4^

 Nanoparticles (NPs) have been designed to imitate or modify biological processes by using nanotechnology in medicine. Solid, colloidal particles of size ranging from 10 nm to ˂1000 nm are defined to be NPs, out of which particles of size less than 200 nm are mostly employed in the biomedical field.^5,6^

 NPs have greater solubility and improved bioavailability owing to their smaller size and larger surface area. They can also cross the blood-brain barrier and quickly absorb endothelial skin cell tight junctions.^7,8^ NPs offer benefits such as hydrophobic stabilization, improved pharmacokinetics and biodistribution as well as targeted drug delivery by reducing toxicity.^9,10^

 The design of nano-formulations involves mixing, adsorption or dissolution of a drug on the surface or in the lipid/polymer matrix. Nanocarriers or particles are classified into two types based on their nature of composition: organic NPs and inorganic NPs. Forms like dendrimers, liposomes, polymeric NPs or micelles and protein-based NPs come under the category of organic NP’s whereas forms like metallic NPs, magnetic and carbon-based nanocarriers falls under the inorganic class of NPs.^11^

 Polymer NPs are recognized by the significant properties of nanocarriers that include nano-size, excellent biocompatibility, biodegradable nature, prolonged bloodstream circulation, enhanced drug loading capability and easy surface change by attachment of ligands. These are promising prospects for diagnosis and treatment for various health aspects such as carcinogenicity.^12,13^ Polymeric nanomedicine further can be categorized into three types– drug conjugates, polymeric micelles and polymersomes.^14,15^

 This article will cover in detail the structure, formulation methods, characterization, biomedical applications, and prospects of polymersomes.

## Structure of polymersomes

**Figure 1:**
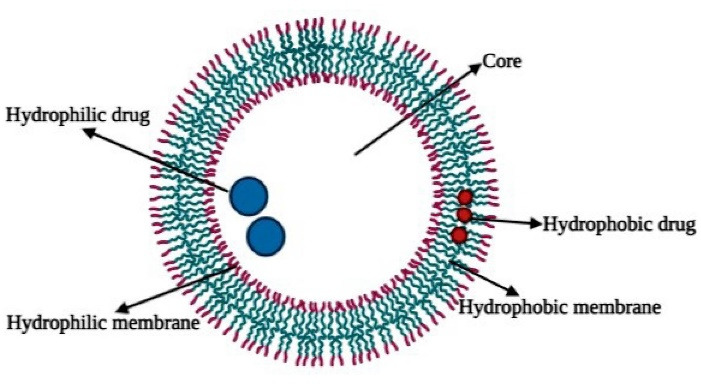
Cross-sectional representation of a polymersome

 Polymersomes are bilayer vesicular systems having hydrophilic–hydrophobic block copolymer with a central aqueous core. This specific structure of polymersomes makes it suitable for both hydrophilic as well as lipophilic drugs. As illustrated in Figure 1, the hydrophilic structure of the interior can be used to embed water solution drugs or agents such as DNA or proteins while the hydrophobic outer bilayers can simultaneously be paired with insufficiently water-insoluble medicines (Figure 1).

 Polymersome dimensions can be controlled through different chemical compositions and synthesis processes as well as solution properties like concentration, temperature and solvent used, etc. Surface modifications like ligand attachment and PEGylation of the polymersomes aid in their effectiveness as drug carriers for anticancer drugs etc.^16-20^

 The mechanical strength of the polymersomes and drug release rate depends on the molecular weight of the polymers. A polymer of low molecular weight can get eroded easily as compared to the polymer with high molecular weight and therefore for achieving the required drug release rate, a combination of low and high weight polymers can be employed. The main driving force for drug release from a polymersome is diffusion which can be affected by factors such as membrane thickness, the diffusion coefficient of drug molecules, and pH of the surrounding environment.^21,22^

 The existence of two different polymer layers i.e., hydrophilic, and hydrophobic portions with different solubility is the driving force for the formation of various types of structures. The amphiphilic copolymers can be placed in water and designed into various forms like spherical, cylindrical, lamellae and vesicular forms. The final morphology of the polymeric structures depends on a parameter known as f- value which is defined as the complete copolymer bulk ratio to the hydrophilic unit. It was found that with the value of “f ” ranging from 25%-40%, it results in the formation of vesicles whereas a value greater than 50% results in spherical micelles.^20,23,24^

 There exists a significant difference between liposomes and polymersomes despite having similar amphiphilic properties. The membrane thickness of polymersomes varies around 5-10 nm which is closer to body cell membrane thickness which is about 8-10 nm. On the other hand, liposomes have a thickness ranging between 3-5 nm. Polymersomes have more similarity with the cell membrane in comparison to liposomes which enhances their compatibility. Liposomes are known for their high permeable nature which also results in a disadvantage of more fluidity whereas polymersomes, because of their inert thermodynamic nature are found to be more stable and have less fluidity, maintaining structural integrity.^12,18^

## Polymers used for the preparation of polymersomes

 Polystyrene based copolymer polymersomes were the first developed forms, which were then replaced by boronic acid derivatives of polystyrene copolymer blocks i.e., PEG-b-PS for delivery of anti-diabetic drugs. Other hydrophobic blocks employed as copolymer blocks in polymersomes synthesis are poly(2- cinnamoyl ethyl methacrylate) (PCEMA), poly(dimethyl siloxane) (PDMS), (polypropylene oxide) (PPO), poly(butadiene) (PBD), etc.^25-27^

 Recent advances have resulted in the utilization of biodegradable polymers such as biodegradable polyesters PEO-b-PLA, polycarbonates such as poly(trimethylene carbonate) (PTMC), poly(fumaric/sebacic acid) and poly(phosphazenes) poly(N-isopropyl acrylamide) (PNIPAM) which are employed in the delivery of drug molecules and large molecules such as small interfering ribonucleic acid (siRNA) and antisense oligonucleotides.^28-30^

 Poly (acrylic acid) (PAA) was the first employed hydrophilic copolymer block. A few others include poly(2-methyloxazoline) (PMOXA), poly(2-methyloxazoline)-b-poly(dimethylsiloxane)-b-poly(2-methyloxazoline) (PMOXA-b-PDMS-b-PMOXA), poly(isoprene) (PI) in poly(isoprene)-b-poly (2-cinnamoylethyl methacrylate) (PI-b-PCEMA), poly (4-vinyl pyridine) (P4VP), poly (4-vinylpyridinum methyl iodide) (P4VPMeI) etc. Other than this, poly glutamic acid (PGA) is used as hydrophilic copolymer blocks such as PTMC-b-PGA(poly(dimethyl aminoethyl methacrylate)-b-poly(l-glutamic acid))(PDMA-b-PGA), PBD-b-PGA etc., were considered for the development of polymersomes as effective therapeutic aid.^31-33^

## Synthesis methods for polymersomes

 The techniques employed for the synthesis of polymersomes depends on desirable size, dispersity range, reproducibility, and high amount of drug loading ability. For promoting maximum encapsulation of the active agent into the polymersome copolymer block composition, charge and interaction between the drug and polymer molecules are to be considered before the development of the formulation technique. Electrostatic interactions and the existence of a hydrogen bond between the polymer and drug molecules mainly act as the driving force for maximum encapsulation of the drug.^34^

 The various techniques employed in the synthesis of polymersomes are discussed thoroughly below and their efficiency, advantages and disadvantages are listed in Table 1.

**Table 1:** Comparison of different methods and their efficient applications

**Method**	**Type**	**Encapsulating efficiency (%)**	**Benefits**	**Drawbacks**
Film rehydration	Solvent-free	3-95%	Simple technique, requires no special equipment, a benefit for synthesizing large molecules due to solvent-free nature.	Results in polymersomes of high polydispersity, the tedious downstream recovery process
Direct hydration	Solvent-free	40-90 %	A short time of preparation (˂1 h), maintains the biological activity of encapsulated molecules	Small scale, not recommended for thermosensitive molecules.
Nanoprecipitation	Solvent- displacement	> 90%	A scalable and rapid method for polymersomes production	Use of organic solvents limited to hydrophobic drugs
Microfluidic	Solvent-free/ Solvent displacement	20-85%	No further post-processing steps are involved, complex structures can be formulated easily, control over polymersomes size	Low productivity, limitations on morphology
Double emulsion	Solvent displacement	50-80%	High encapsulating efficiency for hydrophilic moieties	Tedious process for solvent recovery, denaturation of macromolecules

###  Film rehydration

**Figure 2:**
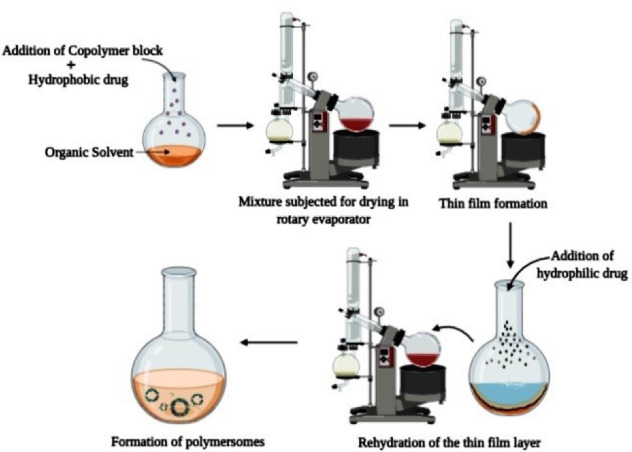
Schematic representation of the film hydration method

 Film hydration is one of the most widely used techniques for the development of polymersomes. This technique, as mentioned in Figure 2, involves an initial step of dissolving the copolymer blocks in an organic solvent such as dichloromethane and dimethyl sulfoxide etc. Then the solution is subjected to drying by placing it in a round-bottomed glass container under vacuum pressure where the polymer solution turns into a thin film layer. The formed residue can be removed by employing a solvent rotary evaporator. Then, the water-based solvent system is to be added to rehydrate the preformed thin film layer and is subjected to continuous stirring.^35^

 The addition of the drug depends on its nature. For example, if a hydrophobic drug is to be encapsulated, it is added at the first step i.e., while dissolving the copolymer in the organic solvent whereas, in the case of a hydrophilic drug, the drug is added to the water phase employed for rehydration of formed thin film layer.^35,36^

 A thin-film rehydration technique has been employed to develop polymersomes for sustained release of doxycycline (DOX), where polyethene glycol-polylactic acid copolymers of different molecular weights were used. The developed polymersomes were found to have 98.08% encapsulation efficiency. In vitro drug release studies revealed that initial burst release i.e., 18%-24% of drug release occurred within 24 hours followed by sustained release of about 40%-60% which occurred for 6 days without any signs of drug leakage. Thus, it can be concluded that the above method resulted in polymersomes of sufficient rigidity and sustain release properties.^37^

 The same technique was also applied in developing PEG-PLGA polymersomes for sustained and controlled delivery of DOX. But in this attempt, the thin polymer film formed was rehydrated by employing ammonium sulphate solution and then was dialyzed against NaCl solution for pH gradient. After this, in presence of continuous stirring, at a temperature of about 60˚C, DOX HCl solution was added. Due to NH_3_ molecules existing outside the vesicle, DOX HCl gets converted into a free form of DOX which passes the hydrophobic membrane and gets accumulated as a gel in the interior portion of the polymersome. The encapsulation efficiency of the polymersomes was found to be 91.25%. In vitro release studies data revealed that polymersomes resulted in 7% sustained drug release for about 5 days with no signs of burst release. A dose of 5 mg/kg weight was administered to healthy mice for evaluating in vivo toxicity of polymersomes and it was found that drug-loaded polymersomes resulted in low cardiac damage when compared to free drugs. Thus, from the above studies, it can be concluded that the method results in polymersomes of high stability and greater therapeutic value. However, the low encapsulating efficiency of the lipophilic drug is a limitation of this technique (Figure 2).^38^

###  Direct hydration

**Figure 3:**
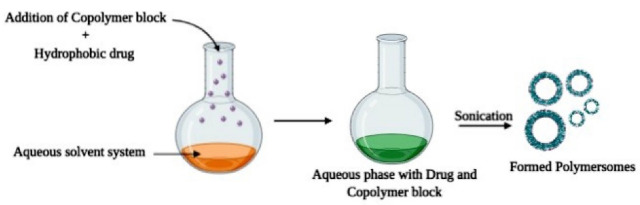
Schematic representation of Direct hydration method

 The direct hydration technique as mentioned in Figure 3 is devoid of organic solvent where the copolymer blocks are directly added to the aqueous phase. First, an aqueous buffer solution is developed based on the nature of polymers and drugs employed and then it is used to dissolve the copolymer blocks where water diffuses to form vesicles. Sonication or freeze-thaw cycle can also be employed to further promote encapsulating efficiency.^39^

 This technique was employed in a study for the development of poly (ethylene glycol)-b-poly (propylene sulphide) nano-polymersomes. The diameter of formed polymersomes was found to be 93.7 nm. Transmission electron microscopy studies revealed the vesicular structure of polymersomes and cellular uptake studies showed 80% uptake of polymersomes by dendritic cells indicating that there exists a vast number of applications of formed polymersomes as drug carriers or in immunization.^40^

 Insulin based polymersomes were developed by using this technique where (dextran-poly(lactodeco-glycolide) was employed as a copolymer block dissolved in phosphate-buffered saline solution. The developed polymersomes were having more than 90% encapsulating efficiency. In vitro insulin release studies were performed in the simulated intestinal fluid of pH 7.4 where 70%-80% of sustained drug release was identified. In vivo anti-glucogenic effect of insulin loaded polymersomes was performed in diabetic rats where reduction in blood glucose levels to 98 mg/dL was observed after 9 hours of post polymersome administration which was sustained for 12 hours. It was found that the formed polymersomes showed greater encapsulating efficiency and provide sustained release of insulin when administered.^41^

 A similar approach was also employed to produce poly (ethylene glycol)-b-poly(caprolactone) based polymersomes where the copolymers were first subjected to melting at a temperature of 60˚C and later distilled water was added to reconstitute. The solution was then passed through a syringe filter to obtain polymersomes of the required size. Like the film rehydration technique, this method also has low encapsulating efficiency of lipophilic molecules as a limitation (Figure 3).

###  Nanoprecipitation

 Often called solvent displacement (solvent switching) or interfacial deposition, nanoprecipitation is the first approach used to encapsulate drug molecules against cancer.^42^

**Figure 4:**
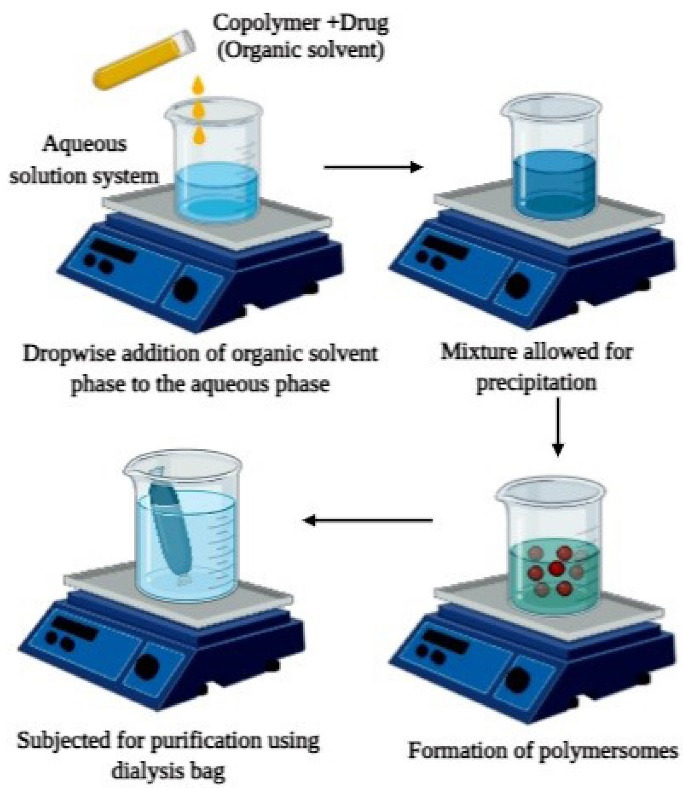
Schematic representation of Nanoprecipitation method

 The technique, as demonstrated in Figure 4, involves the first step of dissolving required quantities of copolymers in a suitable organic solvent which is then added into an aqueous solution system dropwise. The formed solution mixture is then subjected to dialysis to remove the residues of the organic solvent system or any drug molecules which are left unreacted. Centrifuge or reduced pressure can also be employed to remove any traces of organic solvent. The above method is employed in the development of docetaxel loaded polymersomes where dextran-poly (lactic-co-glycolic acid) conjugated with folate is used as copolymer blocks. An organic solution system was first developed by adding the copolymer and drug into dimethyl sulfoxide and the formed solution was then added to deionized water. Traces of dimethyl sulfoxide was eradicated by employing dialysis against water thereby resulting in the formation of polymersomes. The drug loading capacity of polymersomes was found to be 78.85%. When subjected to in vitro drug release studies, initial burst release was seen, the reason of which was found that the drug encapsulated near the surface underwent immediate release which provided the primary dose. Following the burst release, sustained release of about 95% of the drug was found for 6 days indicating the stability of polymersomes and effectiveness as drug carriers.^43^

 A copolymer mixture of poly (g-benzyl L-glutamate) and hyaluronan was used for the synthesis of polymersomes by the above-mentioned technique for encapsulating drug DOX. The drug and copolymers were liquified in an organic solvent which was then added to the aqueous phase i.e., tris-buffer. The traces of organic solvent or drug molecules were removed by employing dialysis. The polymersomes were found to have 50 ± 5% drug loading capacity. In vivo studies for determining the anti-cancer activity of DOX loaded polymersomes were performed on tumor-bearing mice. After one week of therapy, a reduction of tumor size from 80% to 20% was observed, thus indicating that polymersomes developed by the above method can act as carriers for drugs and aids in therapy.^44^ The elimination of the traces of organic solvent was the major limitation of this method (Figure 4).

###  Double emulsion technique

**Figure 5:**
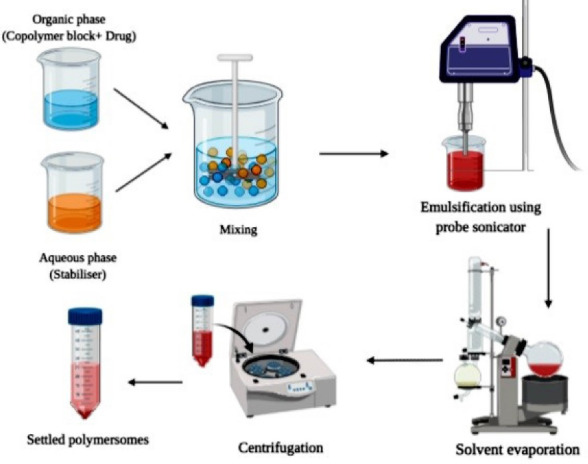
Schematic representation of the Double emulsion method

 This technique involves dissolving the copolymers in a suitable organic phase along with subsequent emulsification of organic phase with aqueous phase (solvent along with a stabilizer or surfactant) as illustrated in Figure 5. The drug molecules are dissolved based on their nature i.e., hydrophilic, or hydrophobic before emulsification. In general, water in oil emulsion is stabilized by size reduction through probe sonication or homogenization. The formed water in the oil emulsion is then further added into a larger volume of the aqueous phase. Organic solvent left could evaporate under vacuum followed by centrifugation. Zhu et al^45^ employed a double emulsion technique for developing ovalbumin (OVA), antigen-loaded polymersomes which provide lymphatic vaccination. Transition electron microscope study revealed that the polymersomes were spherical and have a rough texture. The drug loading content of polymersomes was found to be 118.9 µg/mg. The fluorescent spectral technique was used to determine changes in structural-functional group confirmations, the results revealed that spectra of OVA in water and encapsulated form in polymersomes exhibited similar spectra indicating that the structural integrity was maintained in OVA loaded polymersomes. In vitro antigen release studies have shown that polymersomes result in an initial burst release of about 20% within 24 hours followed by a sustained release for 28 days. Thus, from the results of the above study, it can be concluded that the double emulsion technique results in polymersomes that maintain the structural stability of encapsulated drugs and provide sustained drug therapy. Some of the limitations of this method include the formation of polymersomes of a wide size range and leakage of the drug due to low encapsulation efficiency. The above limitations can be overcome by employing a high shear rate for reduction of size, employing polymers of high molecular weight and concentration, etc (Figure 5).

###  Microfluidic technique

**Figure 6:**
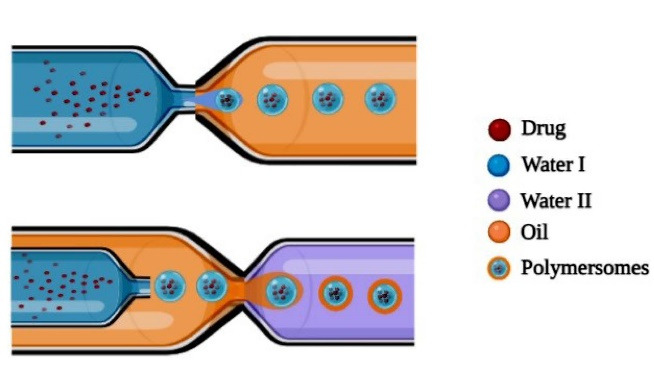
Schematic representation of Microfluidic technique

 Formulation of polymersomes development by microfluidic technique involves two stages: the first stage involves the development of the core segment which consists of aqueous solution and the shell segment comprising of block copolymers in an organic solvent are then converted into core-shell double emulsion droplets by using a microfluidic system. The second stage involves the fabrication of core-shell double emulsion droplets into polymersomes by evaporation of the organic solvent. As mentioned in Figure 6, a microfluidic device is developed for the formation of double emulsions wherein it allows to inject two separate solvent systems thereby resulting in the formation of W/O/W emulsion.^46-48^

 The size of the formed polymersomes ranges between 40 nm to 2 mm which varies depending on the flow rate of solvent systems. Higher flow rate and narrow channel result in the development of smaller sized polymersomes. W/O/W double emulsions composed of PEG (5000)-b-PLA (5000) were formulated by employing microfluidic devices, for encapsulating hydrophilic fluorescent dye in the central shell. The microfluidic device consists of injection syringes where first fluorescence dye-containing droplets are injected followed by middle phase i.e., organic phase comprising of copolymer blocks dissolved in organic solvents like chloroform or toluene are injected which meets the outer phase. At the interphase W/O/W, double emulsion droplets are formed which are stabilized further by evaporating the leftover organic solvent residue.^49^

 Brown et al^50^ developed polymersomes using biocompatible polymersomes like poly(2-(methacryloyloxy) ethyl phosphorylcholine)–poly(2-(diisopropylamino)ethyl methacrylate) (PMPC-b-PDPA) by employing the microfluidic technique. The developed polymersomes were compared with polymersomes developed by the standard solution method for encapsulation efficiency. The polymersomes were incubated within Bovine serum albumin solution for 6 hours. After the incubation period, the amount of drug encapsulated by polymersomes developed by microfluidic technique was found to be 35% whereas polymersomes developed by standard method showed around 21% of encapsulation. Thus, study results reveal that the microfluidic technique results in polymersomes having greater drug-carrying efficiency and thereby aid in therapeutic applications. Thus, microfluidic devices serve as the best aid in the development of highly efficient drug delivery systems for both diagnostic and therapeutic purposes (Figure 6).

###  Supercritical fluid technique

 Supercritical fluid (SCF) of carbon dioxide technology is mostly employed due to its desirable features such as adjustability by changing the temperature and pressure conditions, employing eco-friendly solvents, thereby resulting in polymersomes that are non-toxic and non-flammable. The two-phase system comprising of drug and polymer in cosolvent and SC-CO_2_ mixture at transition point converts into a one-phase homogenous system. Carbon dioxide is the most employed SCF because of its non-toxic, non-flammable and less expensive nature. FDA suggests this technique as safe as it results in nontoxic polymersomes with no residues of organic solvents, thus making this technique the best source for mass production of polymersomes.^51,52^

## Characterization of polymersomes

 Various techniques have been employed for determining the physical, mechanical, and chemical properties of polymersomes and are listed below in Table 2.

**Table 2:** Characterization methods and parametric analysis of polymersomes

**Characterization** **method**	**Size** **detection limit**	**Parameters for analysis**	**Advantages**	**Disadvantages**	References
Scattering Methods: 1. Dynamic Light Scattering 2. Photon correlation spectroscopy 3. X-ray scattering	2 mm 10 mm 0.5 mm	Size, molecular weight, lamellarity, encapsulation permeability, concentration	Simple methods, minimal sample volume required, sensitivity to large molecules, highly accurate results can be obtained	Applicable only for monodisperse sample analysis,chances for misinterpretation, elaborated setup	^53^
Visualization techniques via photons: 1. Optical microscopy 2. Fluorescence correlation spectroscopy	200 mm 200 mm	Size, lamellarity, concentration	Widely employed techniques, better contrast, high sensitivity, Improved resolution	Difficult quantitative analysis, the existence of resolution limit	^29,54^
Visualization techniques via electrons: - 1. Transmission electron microscopy 2. Scanning electron microscopy	0.5 mm-30 mm	Size, morphology, lamellarity, concentration	High resolution, better contrast, structure preservation	Elaborate sample preparation, vesicle structural shrinkage, uncertain values of bilayer thickness and true size	^55^
Electromagnetic manipulation methods: - 1. Scanning probe microscopy 2. Nuclear magnetic resolution 3. Laser doppler electrophoresis	0.1 mm – 1 mm	Size, topology, elastic properties, lamellarity, encapsulation, zeta potential, elastic property, the surface charge potential	High accuracy, fast and high sensitivity	Requires frequent calibration, high sensitivity to vibrations, tedious process	^56^
Sorting techniques 1. Flow cytometry 2. Size exclusion chromatography	50-200 mm	Size, concentration,	Widely available techniques, a wide range of sizes can be studied, more selective	Requires calibration, sample loss occurs due to surface adsorption, elaborate setup, potential pore-clogging	^57^

## Biomedical applications of polymersomes

###  Application in chemotherapy

 Innumerable innovations have resulted in the development of various chemical entities for chemotherapeutical applications where they serve with drawbacks such as toxic side effects, low bioavailability of active drug at the target site, high rate of body clearance, etc., Thus, it is of utmost importance to develop polymersome based drug delivery for enhancing bioavailability, reducing their toxicity, and promoting the effectiveness of anti-cancer drugs.

 Doxorubicin (DOX) is the most widely employed anti-cancer drug but a major limitation associated with this drug was found to be cardiac myocyte toxicity.^58^

 To overcome this toxicity, the chemical entity was transformed into a vesicular form thereby limiting its exposure to cardiac cells. DOX loaded polymersomes were developed by employing amphiphilic copolymers PEO-b-PCL in which poly (ethylene oxide) forms the hydrophilic part and polycaprolactone forms the hydrophobic part. These polymersomes showed hydrolytic degradation. Film rehydration technique was employed for the formation of DOX-loaded polymersomes which were then exposed to two different pH conditions (pH 7.4 and pH 5.5 at 37°C). In vitro drug release kinetics were studied, and it was found that in both the conditions at initial days of exposure burst release of drug was seen. Further exposure to pH 5.5 resulted in a high amount of drug release of up to 80% from polymersomes which was due to acid-based biodegradability of the hydrophobic layer of polymersomes. The in-vivo activity of DOX-loaded polymersomes was studied by administration into mice with a xenotransplant tumor. The drug activity was evaluated based on parameters like tumor size and weight of mice being injected. It was found that the size of the tumor was reduced effectively compared with marketed formulation DOXIL and weight was found to be constant throughout the study period.^59^

 Multidrug treatment for different types of cancer was found to be most effective than individual treatments alone. One such combination is DOX and paclitaxel (TAX). It was found that their efficiency as anticancer therapy was enhanced when delivered together in a vesicular form and it reduced the dose required and cytotoxicity by minimizing exposure to normal cells.^60^

 PEG-b-PLA/PEG-b-PBD copolymer blended polymersomes were developed for delivering a combination of DOX and TAX. The effectiveness of anti-tumor agents was evaluated by injecting the polymersomes into MDA-MB231 human breast cancer cells bearing mice. After few days of injection, the size of the tumor was compared with a control group of mice that received the free drug. It was found that the tumor size was reduced to 60% in mice that were injected with polymersomes and were toxic-free. The tumor suppression resulted within less than 2 days of the span.^61,62^

 Polymersomes were also employed in delivering nucleic acids for cancer therapy. PEG-PLA polymersomes were developed for combined delivery of DOX·HCl and Bc-xL siRNA for effective antitumor activity. It was found that at first, fast release of the drug occurs i.e., DOX from polymersomes followed by siRNA. The efficacy as an anti-cancer therapy was evaluated by treating polymersomes with MKN-45 and MKN-28 cancer cells. It was observed that a high level of apoptosis of cancer cells was seen with minimal doses of DOX and siRNA compared to their free forms.^63^ Thus, it can be found that polymersomes not only result in site-specific drug delivery but also enhances the drug effectiveness by minimizing the rate of clearance from the body.

###  Application in an imaging platform

####  Magnetic resonance imaging 

 Out of all existing forms of imaging techniques, magnetic resonance imaging (MRI) is applied frequently as it provides better resolution, minimal invasiveness etc.^64^ Superparamagnetic iron oxide nanoparticles (SPIONs) are the widely employed MRI agents which serve with the drawback of low solubility in aqueous medium and undergo precipitation in the body.^65^ Formulating the SPIONs in the form of polymersomes results in enhanced stability and tumor cells specificity. Polymersomes were made of biodegradable block copolymer poly (trimethylene carbonate) – poly (L-glutamic acid) (PTMC-*b*-PGA copolymer) and the formed polymersomes were then loaded with SPIONs and DOX HCL which provide a combinational activity of diagnosis and treatment. The resulted particles were found to have minimal particle size thereby resulting in high drug loading efficiency and low rate of clearance from the body and thus providing maximum effectiveness than individual forms.^32^

 Most of the tumor detecting agents have lack specificity and targeting ability. HER2 is one of the most overexpressed antibodies on the surface of tumor cells which can be used as a targeting factor.^66-68^

 Polymersomes made of PTMC-b-PGA copolymers was used as a carrier for SPIONs and drug DOX for both imaging and drug delivery to tumor cells. They were then conjugated with fluorescein/HER-2 antibody which served as an aid for targeting HER-2 expressed on breast cancer cells in mice. It was found that the polymersomes resulted in high contrast imaging along with enhanced apoptosis of tumor cells in mice.^69^

 Pluronic-121 was employed as a polymer for the synthesis of polymersomes which acted as a dual carrier for drug camptothecin and SPIONs for drug delivery to the target prostate cells and imaging. The results were found to be beneficial as it was observed that polymersomes resulted in the reduction growth of tumor cells as well as aided inefficient imaging in the body.^70^

 Ultra-small superparamagnetic iron oxide nanoparticles (USPIONs) serve a dual function as imaging agents and magnetic field generators and are employed in the development of magneto polymersomes. In this, an external high frequency alternating magnetic field is employed as an external stimulus for the drug to get released from polymersomes in a way that is more rapid as compared to drug release through degradation of polymersomes. Polyethylene oxide and butadiene together as copolymers are used for the development of polymersomes with a high pH hydrophilic core for delivering DOX at targeted sites. These are further converted into magneto polymersomes by placing the polymersomes in the solution of iron and are then subjected to electroporation which serves as means for the formation of the porous layer through which magnetic iron particles enter the polymersomes. Thus magneto polymersomes have gained huge attention in the chemotherapeutical and diagnostic file.^71,72^ Thus, from the above illustrations, it was found that polymersomes enhance the contrasting ability and thereby found their use in imaging platforms.

####  Ultrasound technique

 Ultrasound technique of bioimaging gained its significance because of its beneficial outcomes like less cost, easy accessibility, non-invasive nature of imaging and exposure of cells to ultrasound which results in enhanced cellular uptake of drug molecules due to ultrasound-induced cell permeability.^73-75^

 The contrast agents employed in ultrasound are microbubbles filled with gas or air and are of size 3-10 μm. Due to their size, these agents are less stable in blood and have poor tumor tissue penetration. The smaller size and prolonged circulation half-life of polymersomes were utilized for enhancing the imaging activity of microsomes. It also aids in achieving therapeutic efficiency.^76-78^

 In some therapeutic areas, the gas or microbubble formed inside a polymersome through ultrasound can be used as an effective aid in the therapy of tumor cells.

 The above technique is employed for the development of PLGA based [poly (D, L-lactideco-glycolide)] polymersomes filled with H_2_O_2_/Fe_3_O_4_. The hydrophilic core is filled with H_2_O_2_ and the shell is hydrophobically made up of Fe_3_O_4_. When the polymersome structure is exposed to ultrasound, a chemical reaction occurs between H_2_O_2_ and Fe_3_O_4_ which results in the formation of OH free radical and O_2_ therapeutic reactive oxygen species (ROS). The formed elements aids in the treatment and imaging.^73^

 PLGA polymer is employed for developing polymersomes encapsulated with rabies virus glycoprotein peptide for calcium carbonate delivery to neuroblastoma cells. The calcium carbonate, which is generally insoluble at neutral pH conditions, undergo conversion to CO_2_ under acidic conditions. When polymersomes are exposed to tumors, calcium carbonate converts to CO_2 _because of the acidic environment, and it also acts as an effective ultrasound agent. The in vivo results have shown a decrease in tumor size and thus polymersomes serve a dual role of therapy and imaging.^79,80^

 Hydrophilic polyethylene glycol (PEG) was employed as a core-forming agent and poly (L- aspartic acid) was used for providing hydrophobic shell of polymersomes employed in therapeutic delivery of DOX along with diagnostic agent CaCO_3_. When these polymersomes are exposed to ultrasound filed they released DOX which resulted in tumor cell toxicity and CO_2_ acts as an imaging aid.^81^

 In summary, this type of polymersome system containing air together with therapeutic agents may be used as a medical ultrasound device for both imaging and biomedical uses of ultrasonography. Due to the simple preparation procedures and cost-effective equipment, they can be introduced soon as therapeutic and diagnostic tools. Besides, as ultrasound equipment is available in hospitals at low prices, acceptance by the medical community can be greatly increased.

####  Optical imaging

 Optical imaging is one of the most employed techniques as it is less expensive and has high resolution. It employs fluorescent probes which emits near-infrared (NIR) ranged between 700- 900 nm in the wavelength spectrum. One of the drawbacks of this technique is low penetration of the probes into body tissues and degradation in the body. To overcome these limitations, polymersomes are employed as carriers for delivering probes in their functional forms.^82,83^

 Polymersomes made of copolymer poly (ethylene glycol)-*co*-poly (trimethylene carbonate -co-caprolactone) (TCL) were used for encapsulation of multi porphyrin NIR. Fluorophores were employed for bioimaging and were evaluated in vivo in mice bearing carcinoma cells. The results showed that after IV administration of polymersomes, got accumulated in carcinoma cells for about 48 hours with minimal toxicity and more efficacy as optical imaging agents.^84^

 Self-assembly amphiphilic diblock copolymers are employed for developing polymersomes in which they are conjugated with multi(porphyrin)-based NIR fluorophores (NIRF). Study results have shown that polymersomes serve as the best carriers for NIRFs, as they have high loading capacity in their membranes and are structurally stable and unique than liposomes.^15^

 Quantum dots are the most widely employed agents in optical imaging whose efficacy can be further be improved by loading them into polymersomes.^85^ Such a technique was utilized for formulating polymersomes of PEG-PLGA type which contained CdTe quantum dots encapsulated with mercaptosuccinic acid and drug DOX in both core and bilayer. Fluorescence microscopy and tissue homogenate analysis of BALB/c mice with breast adenocarcinoma injected with polymersomes have shown significant size reduction of tumor cells and increased half-life of quantum dots and thereby showing maximum activity.^43^

 Thus, one can utilize polymersomes as carriers for optical imaging agents thereby enhancing its therapeutic applications and minimizing cost.

###  Application as therapeutic delivery agents

 Polymersomes are versatile drug delivery vehicles because of characteristic features like site specificity, structural flexibility preventing leakage of encapsulated constituents, prolonged blood circulation time and longer stability.^86,87^

 Self-assembling polymersomes were developed using hydrophilic polymer hyaluronic acid and hydrophobic fatty acid oleyl amine by ultrasonic method for delivery of vancomycin to treat Methicillin-resistant *Staphylococcus aureus* infections. The developed polymersomes were then evaluated for biosafety by incubating HeLa cell lines with polymersomes for a one-day time duration. The study results have shown 75% cell viability which indicates that polymersomes are safe to use. In vitro drug release studies revealed that an initial burst release followed by a sustained release for about 6 hours from developed polymersomes. In vitro antibacterial studies on *S. aureus* strains resulted that polymersomes have shown antibacterial activity for about 72 hours compared to the free form of the drug. Thus study results signify the advantage of polymersomes as drug carriers because of less toxicity, sustained drug release and long term effectivity.^88,89^

 Matrix metalloproteinase loaded polymersomes (MMPsomes) have been developed which show pH-dependent drug release for treating hepatic fibrosis. A viability assay was performed for determining fewer toxic concentrations which were then subjected to cell fibrolytic studies. It was found that compared to free MMP, the fibrotic marker levels decreased upon treating with MMPsome. Wound healing tests on mice revealed that MMPsomes are effective in wound healing. Further effectiveness was estimated by treating mice with a fibrolytic agent like CCl_4_ and then three days treatment with MMPsomes and free MMP. Study results proved that MMPsomes are most effective agents as anti-fibrolytic than the free form of drug and have more stability as well as enhance the therapeutic effect of the drug.^90^

 PEG-PLGA copolymers were employed for the development of polymersomes covalently conjugated with tetraiodothyroacetic acid for delivering camptothecin to treat colon adrenotumour. The developed polymersomes were then subjected to characteristic studies like transmission electron microscopy and scanning electron microscopy for evaluating surface morphology. Drug loading capacity and encapsulating efficiency of polymersomes was found to be 84 ± 10.12, 4.2 ± 0.82. The polymersomes formulated help in sustained release of drug which was estimated by in vitro drug release studies. Further, when evaluated for cellular toxicity of polymersomes, it was found that they resulted in maximum cell toxicity towards colorectal tumor cells. Their efficacy in treating disease conditions was evaluated in vivo on mice bearing C26 cancer cells. The tetrarch conjugated polymersomes were most effective in reducing tumor cell growth than the drug itself. Thus, the study reveals the significance of polymersomes as drug delivery aids having high efficacy and less cellular toxicity.^91^

 The copolymers dextran and poly (lactic-co-glycolic acid) was employed for the development of amphiphilic polymersomes for intestinal delivery of insulin. The encapsulation efficacy of polymersomes was found to be more than 90%. Phosphate buffer of pH 7.4 was employed as a medium for in vitro evaluation of polymersomes and it was found that the maximum quantity of drug had been released from polymersomes under intestinal pH conditions. Permeability studies of polymersomes were conducted on Madin-Darby canine kidney cell lines. After 4 hours, the permeability was found to be 16.89 ± 0.39%. In vivo studies on diabetes-induced rats were performed where the reduction in blood sugar levels was found to be more as compared to free insulin. Thus, polymersomes loaded with insulin serves as better drug-carrying aids as they have less toxicity but high efficacy.^41^

 The copolymers polystyrene and poly (acrylic acid) were utilized in the development of polymersomes for topical drug delivery of finasteride. The developed polymersomes were surface conjugated with chitosan. Epidermal permeability studies indicated that positively charged chitosan molecules interact with negatively charged phospholipids and result in increased lag time for polymersomes thereby enhancing their topical activity. In vivo drug release studies revealed that greater epidermal permeability was obtained from polymersomes than the free form of the drug.^92^

**Figure 7:**
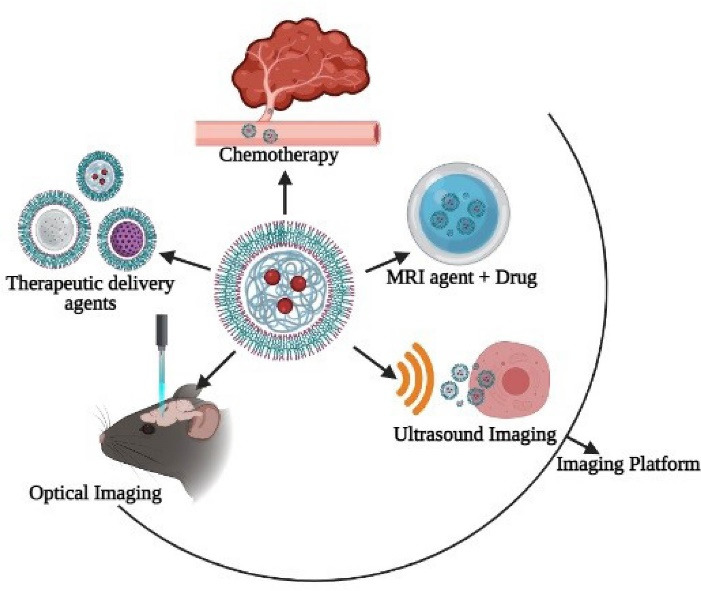
Biomedical applications of polymersomes

 All the applications discussed above are illustrated in Figure 7.

 Over the years diverse carriers have been generated by using polymersomes as a foundation unit or a primary building block and a few of the top listed are mentioned in Table 3.

**Table 3:** Types of polymersomes and applications

**Polymer**	**Application**	**Outcome**	Reference
(PEG-P(TMC-DTC)) And (Mal-PEG-P(TMC-DTC)) Polyethylene glycol-P-Trimethyl carbonate-2,2 dimethyl trimethylene carbonate deblock copolymers	SP94 surface attached polymersomes as carriers for DOX HCL for treating hepatocellular carcinoma	Tumor drug deposition of 14.9% ID/g was observed, polymersomes resulted in a high tumor-to- normal liver tissue ratio of 6.9 indicating its targeted drug delivery	^93^
(PEG- b- PLA) Polyethylene glycol-b-Poly (lactic acid)	Polymersomes for delivery of β galactosidase (β Gal) for enzyme replacement therapy	β Gal loaded Polymersomes of size 147.2 nm were developed, in vivo studies revealed that polymersomes provide 22 times better therapeutic activity than free β Gal.	^94^
(PEG-b-PCL) Poly (ethylene glycol)-b-Poly (ɛ-caprolactone)	Polymersomes for codelivery of Angiostatin and curcumin as antiangiogenics	Polymersomes of size around 140nm were formed, in vitro results revealed that initial drug release of about 59.8% was found within 24 h followed by sustained release of about 67.9% was found after 72 h.	^95^
(M(PEG)-b-DPA-phz) Polyethylene glycol-b-(N, N-di-isopropyl ethylene diamine) phosphazene copolymers	Polymersomes as carriers of IL-2 (Interleukin-2)	IL-2 loaded polymersomes when injected into tumor bearing BALB/c mice have resulted in significant tumor volume reduction from 3600 mm^3^ to 1200 mm^3^ within 16 days which indicates their site-specificity and therapeutic efficacy.	^96^
(PEO-b- PDPA-b- PAA) Polyethylene oxide-b- Poly 2 (di isopropyl amino) ethyl methacrylate-b-Poly Acrylic acid copolymers	Surface Ab labelled polymersomes as delivery agents of DOX and SiRNA for tumor therapy	Polymersomes developed were subjected to in vitro incubation to evaluate cellular uptake of human cervical carcinoma KB cells. The relative fluorescence intensity of KB cells was then measured, and the value was found to be higher i.e., 1.2 RFI which indicates cellular selectivity of polymersomes	^97^
(PEG-b- PLGA) Polyethylene glycol-b-Poly (D, L-lactic-co-glycolic acid)	Lactoferrin surface conjugated polymersomes as a carrier for S14G- human brain delivery	In vivo efficacy of polymersomes for treating glioma was studied by injecting them via intracanal route into CD8 cell bearing BALB/c mice, immunohistochemical staining studies have shown negative staining in the cerebrum and cerebellar regions which indicates significant efficiency of polymersomes	^98^
(PEG-b-PCL) Polyethylene glycol-b- Polycaprolactone	A_665_ and A_666_ labelled polymersomes for targeting outer hair cells of the cochlea	Cochlea hair cell-specific binding of polymersomes was studied in the rat after 12hrs of injection of 6 nmol/mL of polymersomes confocal images revealed that complete uptake by cochlear hair cells was found	^99^
(PBLG-b-HYA) Poly (γ-benzyl-L-glutamate)- b-Hyaluronic acid	Polymersomes as carriers of docetaxel	The size of the resulted polymersomes was found to be 188nm, the polymersomes were incubated with LNC ap cells, after 24 h of study it was found that a complete dose of about 1.50/g was uptaken by the cellular lines.	^100^
(PEG-b-PEO_x_) Polyethylene glycol-b- polyboroxole copolymers	Employed in insulin delivery	Insulin loaded polymersomes upon coming in contact with 0.3M glucose and 0.1 M fructose have shown 50% of insulin release within 1.5 hrs.	^101^
(PEG-b-PCL-b-PDEX) Polyethylene glycol-b- Polycaprolactone-b- phosphorylated dextran	Biocompatible polymersomes for delivery of BSA (Bovine serum albumin), IgG and lysozyme	The polymersomes size was found to be 130-175 nm. MTT assay results revealed that polymersomes are non-toxic up to 0.5mg/ml concentration, in vitro studies have shown that polymersomes delivered proteins into the cytoplasm of RAW 267 cells.	^102^
(PS-PIAT)-b- (PS-PEG-DA) Polystyrene-b-poly c-iso cyanoalanine (2-thiophen3-yl- ethyl amide)-b-Polystyrene-b- poly (ethylene glycol)-oxanor bornadiene copolymers	Polymersomes as carriers of HRP (Horseradish peroxidase), GFP (green fluorescent protein)	Study results provided information on in vitro release behavior and stability.	^103^
(PEO-b-PBD) Poly (ethylene oxide)-b-polybutadiene copolymers	Polymersomes as carriers of TNF-α for prostate cancer therapy	Internalization of polymersomes within prostate cancer cells was found within 24 h of incubation, it was found that 10% of polymersomes resulted in 4.4 folds higher effective anti-tumor activity compared to free drug.	^104^
(PEO-b-PAA-PNI PAM) Polyethylene oxide-b-poly (acrylic acid)-b-poly (N-isopropyl acrylamide)	Polymersomes as carriers of Fluorescein isothiocyanate- dextran	Study results demonstrated the formation of structurally stable and functional polymersomes	^30^
(PMOXA-b-PDMS-b- PMOXA) Poly (2-methyloxazoline)-b- poly (dimethyl siloxane)-b- poly (2-methyloxazoline)	PolyG surface labelled polymersomes for delivery of pravastatin for atherosclerosis treatment	In vitro efficacy of polymersomes was studied using macrophages, after 24 h of incubation it was found that 0.25 µmol/L concentration of pravastatin loaded polymersomes resulted in the decreased cellular activity of macrophages	^105^
(PMPC-b-PDPA) Poly(2-methacryloxy) ethyl phosphorylcholine)-b-poly (2-diisopropyl amino) ethyl methacrylate	Polymersomes for delivery of DNA	Study results revealed that polymersomes developed to serve as potential carriers for macromolecules	^17^

## Existing challenges and clinical breakdown of polymersomes

 Polymersomes despite of their high drug loading capacity, greater stability has certain clinical drawbacks which minimises their utility. In a study conducted, PBD70-b-PEO22 polymersomes labelled with Indium which are of size 90 nm were developed and are studied further for half-life in body fluids using single photon emission computed tomography and was found to be approximately 20 hours. The polymersomes were then compared with PEGylated liposomes of similar size for half-life in rodents, it was found that liposomes were having half life double than that of polymersomes which represents that polymersomes stability in body fluids was less compared to that liposomes.^106,107^ Rapid drug loss due to surface leakage was also found to be one of the drawbacks of polymersomes. In a study where PBD125-b-PEO80 (75 wt%) and poly(L-lactic acid)56-b-PEO109 (25 wt%) conjugated polymersomes loaded with DOX of 3 mg/kg and TAX of 7.5 mg/kg for tumour therapy were developed and the efficiency was studied in tumour bearing mice. Maximum release rate of DOX from polymersomes was found on first day of treatment following which rapid decrease of upto 40% was followed and 0% of drug concentration was found on the fourth day of study. Whereas 0% of TAX traces were found at tumour site during the study interval than the polymersomes compared with market available liposomal dosage form Doxil. It was observed that liposomes resulted in maximum amount of drug release at tumour site for longer duration. Thus the study results indicate that polymersomes are found to have less stability due to rapid surface leakage of drug before reaching target site.^100,108^

 Polymersomes are also employed for oral drug delivery due to their stability in oral fluids, but in a study where PBD-b-PEO polymersomes encapsulating multikinase inhibitor sorafenib were developed for treating hyperammonaemia, where the polymersomes are exposed to bile fluids when orally administered into hyperammonieac rat, despite of having high stability in oral fluids polymersomes were found to be less stable towards biliary secretions resulting in minimising the drug release. Polymersomes developed when compared with market available oral hydrogel of sorafenib found to be less effective which represents that further more clinical studies of polymersomes have to be conducted in bile fluids to determine their stability and effectivity.^90,109^

## Future perspectives

 Nanocarriers face current problems of low cell penetration and inefficient intracellular transport. The numerous design features of polymersomes including the structure of polymer boulders in general, polymer topology, ability to shape in spherical or non-spherical vesicles and membrane thickness, the ability to act as a carrier for hydrophobic or hydrophilic drugs and so on have contributed to their flexibility and tunability. Despite major changes, polymersomes are yet to enter clinical trials, including loading performance, high-process preparedness methods and multifunctionality and success in pre-clinical assessments (in vitro and in vivo). Its clinical translation would require ensuring their safety be concentrated using biodegradable non-toxic polymeric materials. To this, more energy needs to be invested in the production of carriers created by reproducible methods of formulation, using entirely biocompatible polymers, the knowledge of biological existence, the leverage of endosomal escape engineering and biologic interactions. The polymersome clinical trials remain many years away but the researchers are concentrating on targeting and imaging cell types using animal models. In general, the production of polymersomes to be spatially and temporally regulated with the transport, safety, aiming and release of chemical therapeutic agents is ongoing and will provide flexible use of the product from an application perspective. Polymersomes are useful for the interpretation of cancer tissues using contrast or fluorescent material. This technique can be used to capture pictures of deeply seated tumors and helps in targeted drug delivery.

 Polymersomes are thus highly special and are used in a wide variety of biomedical applications. This offers tailored care which can revolutionize current therapies.

## Conclusion

 Polymersomes are versatile systems that offer a multitude of advantages that enhance their applicability clinically and therapeutically. Polymersomes encapsulate hydrophilic, hydrophobic, and amphiphilic molecules like any other vesicular structure, permeability of polymersomes aids in controlled release of contents loaded following its thick and robust membrane provides superior stability in vitro and possibly in vivo. This review summarizes various synthesis techniques for the development of polymersomes each of its owing advantages followed by disadvantages, and characterization techniques of developed polymersomes. Polymersome based drug delivery for enhancing bioavailability, reducing their toxicity, and promoting the effectiveness of anti-cancer drugs. Structural properties results in developing multi-drug loaded polymersomes for tumour therapy. Due to the direct delivery of the active moiety in the diseased tissues/organ, the cytotoxicity of several therapeutic agents was decreased and enhanced therapeutic efficiency. Polymersomes polymersomes enhance the contrast ability of imaging agents thereby found its use more in imaging techniques like MRI, Ultra sound technique and optical imaging. The demarcation line between diagnosis and treatment has been broken in functional polymersomes in theragnostic applications. Therapeutic applications of polymersomes developed using various types of polymers are mentioned in this article. Polymersomes can therefore be inferred as new and onward therapeutic systems for safe and successful treatment of major health problems and improvising standards of treating various diseases.

## Acknowledgments

 Authors would like to thank Manipal College of Pharmaceutical Sciences, MAHE, Manipal, India. Authors are also thankful for Biorender.com, an image-making tool.

## Competing Interests

 The authors declare no conflict of interest.
